# Updates on Aptamer Research

**DOI:** 10.3390/ijms20102511

**Published:** 2019-05-21

**Authors:** Mohamed H. Ali, Marwa E. Elsherbiny, Marwan Emara

**Affiliations:** 1 Center for Aging and Associated Diseases, Zewail City of Science and Technology, Giza 12578, Egypt; mali@zewailcity.edu.eg; 2 Department of Biochemistry and Cell Biology, Stony Brook University, Stony Brook, NY 11794-5215, USA; 3 Department of Pharmacology and Toxicology, Ahram Canadian University, 6th of October City, Giza 12566, Egypt; marwae@ualberta.ca

**Keywords:** aptamer, SELEX, antibodies, nanobodies, super-resolution microscopy

## Abstract

For many years, different probing techniques have mainly relied on antibodies for molecular recognition. However, with the discovery of aptamers, this has changed. The science community is currently considering using aptamers in molecular targeting studies because of the many potential advantages they have over traditional antibodies. Some of these possible advantages are their specificity, higher binding affinity, better target discrimination, minimized batch-to-batch variation, and reduced side effects. Overall, these characteristics of aptamers have attracted scholars to use them as molecular probes in place of antibodies, with some aptamer-based targeting products being now available in the market. The present review is aimed at discussing the potential of aptamers as probes in molecular biology and in super-resolution microscopy.

## 1. Introduction

Nucleic acids (NAs) were for a long time considered compounds whose major functions were related to the storage of inherited information (DNA) and its transfer from gene to protein through RNA [1,2]. However, studies have over time confirmed that NAs perform other functions such as enzymatic catalysis and the regulation of transcription. The discovery of these additional functions of NAs has compelled the scholarly community to reconsider its original position concerning the functions of NAs [1]. Consequently, this change has led the scientific community to propose the “RNA world theory” [3,4,5]. In particular, this theory posits that NAs possess the ability to perform multiple functions and have presumably facilitated all the catalytic reactions that started life on earth [4,6]. The discovery of aptamers is indeed a valuable contribution towards the empirical determination of the multifunctional nature of NAs [1,7]. Essentially, aptamers are small, often ranging between 20 to 60 nucleotides, single-stranded DNA or RNA oligonucleotides that are capable of binding target molecules not only with high specificity but also with high affinity [8,9,10,11,12]. 

**Figure 1:**
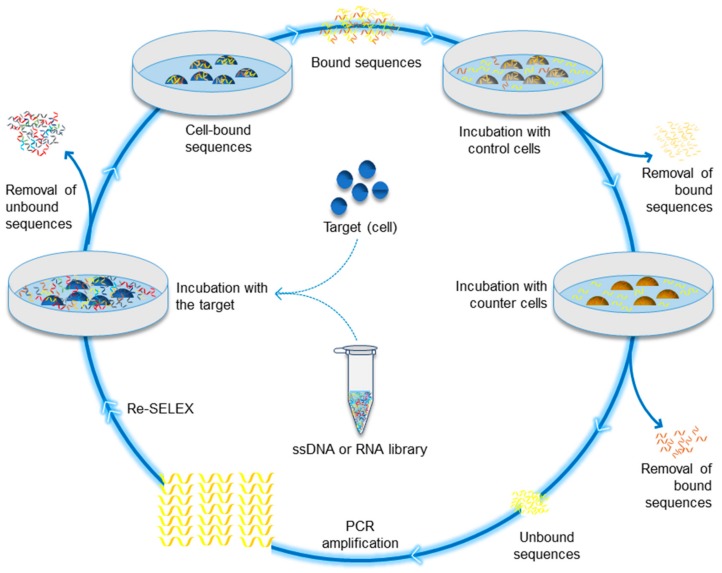
Cell-SELEX aptamer selection method. It consists of three major steps that can be interspersed with sub-steps: a. preparation of the ssDNA or RNA library, b. incubation of the target molecules with the library as a positive selection step, and c. amplification of the selected and recovered strands. Some sub-steps can be performed, whether before or after the positive selection, such as incubation with counter cells in order to remove the non-specific binding and enhance the selection process.

Aptamers, the output of systematic evolution of ligands by exponential enrichment (SELEX) process, have been available for about three decades. In particular, the development of aptamers is attributed to the work of Craig Tuerk and Larry Gold in 1990 [13]. Gold and Tuerk focused on investigating the nature of the “translational operator” within the bacteriophage T4 gene 43 mRNA. This research led to performing the first SELEX experiment that produced two winning hairpins, the wild-type T4 sequence as well as another containing quadruple mutations, both bound to the gene 43 protein with the same affinities [13,14]. Although, Tuerk’s experiments pioneered the SELEX process, the process was not named SELEX until Szostak and Ellington developed the same method and coined the term SELEX [15]. The term refers to a process that entails progressive separation of single-stranded DNAs or RNAs (ssDNA/RNA) combinatorial single-stranded oligonucleotide library via repeated rounds of binding, partitioning, and amplification [16,17,18]. It is noted that following the development of this novel technology, different modified SELEX techniques have emerged over the past three decades. Table 1 summarizes the different SELEX methods, as described in their original context. Some of these developments tackled the ssDNA/RNA library improvement, while many others addressed the development of the SELEX process. In the contemporary research environment, cell-SELEX (illustrated in Figure 1) has been extensively used to identify and select aptamers that can help in the diagnosis as well as the development of treatments for different diseases, particularly for cancer [18,19,20]. Thus, cell-SELEX technology has become increasingly important in medical research. 

Currently, a significant number of generated aptamers are indeed capable of binding different targets, including large protein complexes, simple inorganic molecules, and whole cells [10]. From this prospective, aptamers are simply considered nucleotide analogues of antibodies [11,106,107]. However, compared to antibodies, the generation of aptamers is significantly easier and cheaper. Thus if appropriately developed, these characteristics make aptamers ideal candidates for a wide variety of applications such as disease diagnosis, biosensor design, nanotherapy, molecular imaging, and the purification of particular target molecules from complex mixtures [9,10,108].

The science community has in the recent years developed super-resolution imaging techniques that offer three-dimensional imaging capabilities that overcome the diffraction restrictions of conventional light microscopy especially in viewing lateral dimensions [109,110]. For example, techniques such as single-molecule localization or stimulated emission depletion microscopy (STED) and secondary ion mass spectrometry microscopy have enabled scientists to visualize whether objects are in fact localized [111,112]. However, the advancement of these techniques requires additional improvements in the fluorescent labels to harness the power of super resolution as the image resolution is also restricted by the effectiveness of the labelling probes’ size, specificity, stability, and density [113]. Indeed, the development of these novel techniques has encouraged researchers to reconsider labelling in super resolution. Aptamers’ favourable characteristics have attracted scientists to consider using them in place of antibodies in super resolution. The focus of this review is to demonstrate the potential of aptamers as probing tools that can be used in super resolution. 

## 2. Antibodies and Aptamers

Antibodies, as pointed out in the preceding section, are a popular class of compounds that have long been used in research to provide molecular identification for a broad array of applications, including disease diagnosis and therapy. Consequently, antibodies have made significant contributions towards the improvement of diagnostic assays [114,115]. In fact, it can be argued that antibodies have become increasingly indispensable in most of the clinical diagnostic tests in the modern medicine. However, the emergence of SELEX (outlined in Table 1) has made it possible for researchers to progressively isolate oligonucleotide sequences capable of recognizing nearly any class of target molecules not only with high affinity but with high specificity as well [115]. Although completely distinct from antibodies, aptamers are thought to emerge as a class of compounds that rival antibodies in terms of both diagnostic and therapeutic applications [116]. Indeed, the available body of knowledge has confirmed that this class of molecules mimics the properties of antibodies in several formats. Mairal et al. contended that the high demand for diagnostic assays that can effectively help in the management of present and emerging diseases has soared in recent years and it is suggested that aptamers could potentially satisfy various molecular recognition needs in these diagnostic assays [114]. Although the research on aptamers is still in its infancy, there is compelling evidence that this field of research is progressing at a fast pace [117]. 

The discovery of aptamers has already helped circumvent some of the key limitations associated with antibodies [118]. A critical evaluation of the present body of knowledge on antibodies and aptamers shows that these classes both have advantages and disadvantages (summarized in Table 2) over each other with multiple pieces of empirical evidence reveal that aptamers provide a broad array of clear-cut merits over conventional antibodies [115,119,120,121]. 

Some of the key advantages of aptamers include their inertness towards the surrounding cells [114,122,123]. Specific aptamers are capable of binding to a target molecule with high precision and, thus, it is thought that this can facilitate a broad array of diagnostic and therapeutic applications without the fear of potential non-specific binding [114]. Another advantage is that they often have a higher binding affinity [123,124]. It is indeed acknowledged that in cases where several ligands can bind to the same receptor site, aptamers have been found to offer a relatively higher binding affinity. It is worth noting that, in practice, the higher the affinity level of a biological molecule used in molecular recognition, the less quantity is needed in molecular identification, which may reduce the costs of performing molecular identification studies [122,125,126].

Aptamers also often exhibit excellent target molecule specificity compared to antibodies [123]. According to Kedzierski et al., there are aptamers that exhibit >10,000-fold binding affinity for theophylline over caffeine [123]. There is no doubt that this excellent target specificity makes aptamers ideal bio-markers as they increase the accuracy of molecular recognition. Another advantage is that aptamers facilitate the discovery of unknown biomarkers [123,127]. Kedzierski et al. explained that different SELEX techniques are capable of identifying unknown biomarkers and consequently, this has in the recent years hastened the discovery of diseases and therapeutics [123]. Some other advantages of aptamers over the conventional antibodies include discovery time savings, minimized batch-to-batch variation, in vitro vs. in vivo testing advantages, increased stability, and reduced side effects [123].

It is worth noting that nanobodies, antibody-mimicking binders that are a single variable domain of an antibody, are also capable of specific binding and are also used in molecular targeting [128]. Previous studies have suggested that the use of affinity probes such as aptamers and nanobodies has many advantages in light microscopy, particularly in the field of super resolution [129,130]. De Castro et al. explained that steric hindrance causes less impairment on these small molecular probes and, thus, facilitates their penetration into biological samples and their binding to epitopes that are traditionally inaccessible to larger antibodies [129]. Indeed, traditional light microscopy suffers from limited resolution capabilities due to light diffraction [113,131]. Many methodologies that are capable of overcoming this limitation were developed in the recent years [113]. In fact, it has been reported that diffraction-unlimited microscopes are improving rapidly and, as a result, it is now practical to attain excellent resolutions of small elements of less than 10 nm [132]. Nevertheless, the enhancement of sample preparation and methodologies used in staining is still lagging behind [39]. The conventional immunostaining technique largely depends on affinity tools that at times are larger than the protein of interest making it impossible to fully exploit the potential of contemporary imaging techniques [129,133]. However, it is anticipated that small probes such as nanobodies and aptamers would have a significant impact on the precision of staining biological samples [129]. Recent studies have in fact suggested that when compared to traditional antibody techniques, nanobodies are capable of positioning the fluorescent molecules closer to the intended target, and thereby resulting in enhanced localization precisions using super-resolution microscopy [134,135]. Likewise, in a comparative study of aptamers vs. antibodies that involved STED, it was concluded that in super-resolution microscopy, aptamers offer superior staining of different cellular receptors. Since aptamers are cheap to select and nanobodies usually have higher affinities, it is thought that aptamers and nanobodies complement each other and together they should help advance the effectiveness of super-resolution imaging [136].

Although the use of aptamers as molecular identification compounds has emerged as a viable approach for diagnostics, therapeutics, and biosensing [124], aptamers do present a set of challenges that have inadvertently hampered both their research and commercialization. Firstly, relatively few aptamers bind small molecules [124]. It is argued that small molecules are crucial targets for research because of their clinical and commercial uses as well as diverse biological functions. Therefore, the availability of relatively few aptamers binding to small molecules restricts research on their biological functions, clinical use, and commercial use. Secondly, aptamers are likely to encounter rapid clearance rate from the circulation because of their small size as well as degradation by nucleases especially for unmodified aptamers [123]. Further, there are examples where developing a selective aptamer still represents a challenge. For example, aptamers that were selected against monomeric/oligomeric forms of amyloid peptide had very low affinity to these forms, although they exhibited a strong non-specific affinity to amyloid high-molecular weight fibrils [137,138]. It is thought, however, that aptamers still hold promise for the treatment of Alzheimer’s disease because current amyloid inhibitors and antibodies are not effective at the membrane surface, where inhibition of amyloid aggregate and channel formation is needed to stop disease progression [139]. Janas et al. 2019 shows that functional exosomes containing the selected pool of aptamers can inhibit the formation of amyloid aggregates and channel activity at the membrane surface [139].

Nevertheless, aptamer microarray technology has recently been developed in an effort to overcome the drawbacks and limitations of antibody microarrays, especially when targeting small molecules with one epitope [140]. Indeed, aptamers that target small organics, peptides, proteins, viruses, cells, and bacteria are now commercially available from a number of emerging companies such as Aptagen and AMSBIO. Other companies have been established focusing on aptamers-based drug discovery such as OSI Pharmaceuticals, Nascacell Technologies in collaboration with Discovery Partners International, and Aptanomics. In addition, aptamer-based clinical trials are currently being run by different companies such as Baxter Biosciences, Corgentech, Bristol-Myers Squibb, Gilead Sciences, NOXXON Pharma, Oxford Cancer Research, Pfizer, and Regado Biosciences (reviewed in [141,142,143]), while others are developing aptamer-based diagnostics or therapeutics including Aptamer Group, Base Pair Biotechnologies, and Ophthotech Corporation [144].

## 3. Super-Resolution Imaging

For many years, light microscopy has significantly improved the scientific understanding of how cells function [113]. In fact, the different spheres of biology are believed to have developed from images that have been acquired under light microscopes. Nonetheless, studies that utilize conventional light microscopy techniques have been limited to the resolution of about 200 nm [113]. The science community has for many years focused on developing several imaging techniques to address the diffraction of light [145]. Some of these imaging techniques include multiphoton fluorescence microscopy and confocal microscopy. Fundamentally, these novel imaging techniques have not only significantly enhanced the resolution of cellular images, but have also reduced the out-of-focus fluorescence background [113]. Consequently, this has facilitated optical sectioning and three-dimensional imaging. Although these techniques have significantly improved resolution, the approaches are still limited by light diffraction [146]. In practice, these advanced techniques have often achieved resolutions of ~100 nm in all three dimensions [113,146].

Recently, this limitation in light microscopy has been overcome by many improved microscopy techniques, with some of the techniques achieving resolutions of ~10 nm [130]. The capability of these novel advanced microscopy techniques to circumvent light diffraction has significantly improved imaging precision [113]. Consequently, this has enabled researchers to identify important cellular details that could not be isolated using the traditional diffraction-limited instruments. STED, single-molecule localization techniques (SMLM), positron-emission tomography (PET), single-photon emission computed tomography (SPECT), and cryo-electron microscopy (cryo-EM) are some of the common diffraction-unlimited techniques that have made a significant impact in biological sciences [147,148,149,150,151]. For example, stochastic optical reconstruction microscopy (STORM), an SMLM technique, offers a high spatial resolution for the position of the fluorophore [152]. It is worth noting that large labels, for example, antibodies, can provide a misleading position of the fluorophore from the target molecule [153]. However, the discovery and subsequent use of different SMLM techniques made it possible to accurately pinpoint the position of the fluorophore from the target molecule. 

In principle, STED utilizes pairs of synchronized laser pulses, which play a critical role in overcoming the challenge of light diffraction [154]. The basic functioning mechanism of this super-resolution microscopy technique is that it creates images through selective deactivation of fluorophores [155]. This deactivation reduces the illumination area at the focal point and thus improves the achievable resolution for a specific system.

PET has also remarkably revolutionized the field of super resolution, particularly in biomedical research [91,93,94]. This advanced imaging technique utilizes small amounts of radiotracers, a unique camera, and a computer to help in the evaluation of tissue and organ functions in patients [156]. It is actually possible to detect early onset of diseases by identifying changes happening at the cellular level using PET [130,157,158,159]. 

Finally, cryo-EM is another super-resolution technique that is used to image frozen hydrated specimens at cryogenic temperatures [160,161]. This imaging technique allows the specimens used in super-resolution studies to remain in their native state [161]. Thus, this enables the study of fine cellular structures, protein complexes, and viruses at molecular resolution [162].

## 4. Aptamers for Super-Resolution Imaging 

The enhanced imaging precision that resulted from the development of the previously discussed imaging techniques has also revealed that traditional staining using large affinity tags, for example, antibodies, are not sufficiently accurate [130]. It is argued that since aptamers are significantly smaller than antibodies, these molecules might provide a practical advantage in super-resolution imaging [129,130,163].

In their study, de Castro et al. particularly compared the live staining of transferrin receptors acquired with varied fluorescently labelled affinity probes, including the natural receptor ligand transferrin, specific monoclonal antibodies, or aptamers [130]. Analysis of the collected data showed that there were insignificant variations between the three mentioned staining strategies when imaging is carried out with traditional laser scanning confocal microscopy. Nevertheless, the aptamer tag showed clear superiority over antibodies in the super-resolved images acquired with STED microscopy. Thus it was concluded that compared to the natural receptor ligand transferrin and specific monoclonal antibodies, aptamer staining is ideal in super-resolution microscopy [130]. It is worth noting that similar observations were reported when aptamers were compared to antibodies targeting different epitopes including prostate-specific membrane antigen and EGFR [18,129,136,164].

In another study that targeted three membrane receptors that are relevant to human health and cycle between the intracellular space and the plasma membrane, de Castro et al. observed that, compared to the majority of antibodies, aptamers were capable of revealing more epitopes and thus, offered denser labelling of stained structures, which enhanced the quality of the information obtained from the images [129]. In this study aptamer labelling was advantageous under both super-resolution imaging and light microscopy imaging. 

EGFR aptamer labelling also achieved a better quality in dSTORM imaging. In a recent study, aptamer labelling achieved detailed and precise structural analysis of the active EGFR that forms large clusters compared to EGFR at rest. This active-to-rest EGFR structure difference has not been detected using traditional antibodies [163]. The ability of aptamers to detect small structural changes makes them promising candidates for studying the morphological changes occur for the membrane proteins during different biological activities [163].

Additionally, in vivo PET scanning of BT474 mice showed higher uptake of ^18^F-labelled aptamers that specifically bind to breast cancer expressing HER2 [165]. The confocal images also confirmed the specificity of ^18^F-labelled aptamers to HER2-positive cells [165]. For strategies employed in the development of aptamer-based SPECT and PET imaging we refer the readers to an informative review by Hassanzadeh et al. [166]. 

Aptamers are particularly appropriate structure-changing molecules that are capable of combining highly selective biorecognition as well as signal transduction [167]. It is possible to select aptamers towards nearly any ligand molecules through SELEX and can readily be incorporated into the functional devices that boost new applications that range from DNA-machines in sensors to the bioseparations. It has been reported that binding of ligands often affects the stability of the nucleotides that the ligand directly contacts [168]. Ligand binding also causes distal rearrangements in the structure of aptamers. These structural changes can easily be observed by evaluating the cleavage patterns using cryo-EM. Aptamer-based labelling need to be done before the freezing step [162]. In their study, Stanlis et al. showed that ssDNA aptamers could be a useful tool for achieving accurate localization of specific proteins, under freeze-substitution fixation conditions, during studying the cellular fine structures using cryo-EM [169]. They indicated that aptamers are sufficiently soluble probes to be used along with the organic solvents used in freeze-substitution conditions; however, it failed to achieve high-affinity binding [169]. 

Apart from possessing unique physical properties that make them superior to the conventional antibodies in super-resolution imaging, there are several particular modifications that allow aptamers to achieve the highest possible resolution. Choosing an acceptable photostable probe is one of the key features dictating the super-resolution imaging quality [170,171,172]. Using stable tagged-aptamer-based probes in super-resolution microscopy allows obtaining favourable signal-to-noise ratio and also localizing the probes to specific targets without being degraded by the cellular machinery. One particular approach to achieve acceptable aptamer stability is the use of Spiegelmer technology. For example, instead of endonuclease-sensitive RNA aptamers (D-form), researchers select endonuclease-resistant RNA aptamers (L-form) that result from binding to the mirror-image of the intended target molecule [173,174,175,176]. The mirror-image aptamer (L-form) subsequently is expected to bind the natural target molecule [173,177]. Additionally, the substitution of the natural D-ribose with L-ribose renders the mirror-image aptamer completely stable. Another strategy that can be used to enable aptamers to achieve better resolution is to optimize the density of the target molecule and similarly the concentration of the library, that would increase the signal (target-aptamer interaction)-to-noise (interfering species) ratio [174,178,179]. 

The discovery of aptamers presents an opportunity for biomedical researchers to develop novel molecular targeting probes and effective diagnostic/treatment tools for different diseases. Undoubtedly as well, these molecules are promising tools for super-resolution microscopy and have already been shown superior to conventional antibodies in some applications.

## Figures and Tables

**Table 1:** Important strides in SELEX research.

SELEX Type	Principle	Aim	Application/Result	Year	Ref.
Negative SELEX	In vitro selection of single-stranded oligonucleotides against a target after removing sequences that bind to partitioning/immobilization matrices	▪ Remove false-positive sequences selected against the partitioning matrices ▪ Increase SELEX affinity by preventing non-specific interaction	Isolated ligand-specific aptamers that selectively bind to small molecules (dyes)	1992	[21]
In vivo SELEX	The selection and amplification steps occur inside the living cell using retroviral-based replication system	Produce aptamers that are functional in vivo	Provided a method to transfer aptamer selection and amplification from in vitro to in vivo	1993	[22,23]
Counter SELEX	Uses a second elution step against a molecule of similar target structure (e.g., caffeine, which differs from theophylline at the N-7 position)	Increase aptamer’s specificity and affinity towards target molecules	Isolated aptamer of high-specificity to theophylline	1994	[24]
cDNA-SELEX	Uses a preselected natural oligonucleotide pool that binds to the protein of interest rather than using a synthetic library	Identify natural library that interact with the protein of interest	Provided a new approach to study the interaction between the nucleotides and the protein of interest	1995	[25]
Blended SELEX	Forming a blended-pool through linking molecules (e.g., inhibitor) to a specific site on the library that will be subjected to SELEX	▪ Obtain a highly active and specific variable of the bound sequence through incorporation of functional non-nucleic moieties ▪ Obtain aptamers toward a specific site of the target	▪ Provided a new strategy to design the aptamer toward different moieties that can be attached to the library ▪ Provided alternative way to enrich the target pools required for drug discovery	1995	[26]
▪ PhotoSELEX ▪ Photo-crosslinking	Uses photoactivatable aptamers to crosslink and/or photoactive the target	▪ Select aptamers of high-sensitivity, affinity, and specificity ▪ Photo-induced crosslinking allows rigorous washing	▪ In Vitro selection of aptamers that can photo-crosslink HIV-1 Rev protein and human basic fibroblast growth factor	1995 2000	[27,28]
Spiegelmer^®^	Selection of D-oligonucleotides against a mirrored target. Then, the D-oligonucleotides will be synthesized as L-oligonucleotides that can bind to unmirrored target	Increase aptamer stability against nucleases	Identified an endonuclease-stable L-RNAaptamer that binds to D-adenosine	1996	[29]
Magnetic bead-based SELEX	Uses magnetic beads for immobilization	▪ Use small amount of target ▪ Achieve easy and quick separation of protein‒aptamer complex from the free oligonucleotides	Provided a new method to avoid DNA precipitation	1997	[30]
EMSA-SELEX	Uses EMSA partitioning at every cycle of the selection process	Identify a method to study oligonucleotides binding to proteins	Identified the role of specific zinc finger motifs in the Roaz protein that help in DNA‒protein binding and protein‒protein interaction	1998	[31]
Cell-SELEX	Identifies aptamers that bind specifically to different cells or targets on cell membrane	▪ Design high-affinity aptamer towards any single protein ▪ Identify aptamers specific for cell membrane targets	Identified aptamers that binds to specific cell (e.g., RBCs) or cell surface protein (e.g., variant surface glycoprotein of Trypanosoma brucei)	1998 1999	[32,33,34]
Chimeric SELEX	Fuses pre-selected aptamers of different targets together to form a combined small library followed by applying a dual selection pressure to select aptamers that can bind to multiple targets	Generate dual-function aptamers	Generated an engineered dual-function aptamer capable of testing/binding to two distinct features/targets	1998	[35]
Multi-stage SELEX	Modified chimeric-based SELEX followed by an extra selection with entirety of targets	Develop an allosteric synthetic aptamer	Provided a method to study the allosteric interactions in the DNA	1999	[36]
Indirect selection	Aptamer binds to a “primary target.” Binding to this target is required for aptamer bind ing to the main target	Develop a transition-state-bound aptamer that facilitates its binding to the main target	Generated Zn^2+^-dependent aptamer molecule for specific target binding to HIV-1 Tat protein	2000	[37]
Toggle-SELEX	A “polyclonal” aptamer selection strategy that generates aptamer capable of binding to multiple proteins through incubating the pre-selected aptamer with a second target until the aptamer can identify another region for binding	Select aptamers against homologous targets depending on cross-reactivity	▪ Selected in vitro “polyclonal” aptamer that can bind to human and porcine thrombin ▪ Provided a cheap method to produce aptamers for animal testing	2001	[38]
▪ Truncation SELEX ▪ Primer-free genomic SELEX ▪ MP-SELEX (Primer-bridge PCR) ▪ PF-SELEX (self-bridge RT-PCR)	Truncates fixed regions present in the aptamers sequences that may limit the structure variations or reduce the ability of aptamer binding to the target	▪ Generate aptamer with minimized or eliminated fixed sequences ▪ Avoid nonspecific binding ▪ Avoid chemical modification	Provided a method to control aptamer-fixed sequences	2001 2004 2008 2010	[39,40,41,42]
Expression Cassette SELEX	Fuses a pre-selected aptamer with a polymerase III promoter “expression cassette” in a DNA plasmid	Produce highly expressed levels of functional aptamers	▪ Provided a method to increase aptamer expression ▪ Produced stable aptamers	2002	[43]
NECEEM	Uses non-equilibrium conditions to separate the constituents of protein‒DNA mixture (free and bound components)	▪ Produce high separation efficacy ▪ Study macromolecular interaction	Provided a novel electrophoretic method for studying protein‒DNA interaction and *K_d_* of the DNA‒protein complex	2002	[44]
Subtractive SELEX	Similar to counter SELEX where it adopts extra SELEX rounds to control target binding	Distinguish cell subtypes of homologous origin	Distinguished between differentiated and normal PC12 cells	2003	[45]
Tailored SELEX	Isolation of short aptamer through designing of cleavable sites to remove fixed sequences	▪ Produce short aptamer sequence without the need for primers ▪ Avoid post-SELEX truncation	Provided a new method to identify short fixed nucleotides aptamers through ligation and removal of primer	2003	[46]
CE-SELEX	Uses differential electrophoretic separation at every SELEX round after aptamer binds to the target	▪ Reduce the selection rounds ▪ Decrease the selection time	Standardized the SELEX method and made it more efficient	2004	[47]
SweepCE	Uses protein non-stopped flow in the capillary electrophoresis to form DNA‒protein complex that causes sweeping of the DNA	▪ Produce faster aptamer-target complex formation ▪ Measure the rate-constant of complex formation	Provided a method that help in studying the equilibrium and kinetic parameters during protein‒DNA complex formation	2004	[48]
On-chip selection	On-chip selection method combined with point-mutation of the sequence and usage of genetic algorithm to produce aptamers	Generate different aptamers against different target molecules	Generated aptamers against resorufin	2004	[49]
ECEEM	Maintains the equilibrium of the aptamer-target while separating the components of the equilibrium mixture by capillary electrophoresis	▪ Produce alternative way for separation of aptamers with specific predefined *K_d_* ▪ Categorize different aptamers based on their *K_d_* ▪ Reduce the selection rounds ▪ Decrease the selection time	Produced “smart aptamers” with a predefined *K_d_* value matching the theoretically predicted value	2005	[50]
FluMag-SELEX	Magnetic beads-based method using fluorescent labelling for monitoring the affinity and complex separation	▪ Rapid separation of bound and unbound molecules ▪ Avoid use of radioactive isotopes-labelled libraries	▪ Provided a new method for selecting a target of diverse properties and sizes and linking it to magnetic beads that improved the selection specificity ▪ Provided a way to use the aptamers as biosensors	2005	[51]
Non-SELEX	NECEEM-based method in which the PCR amplification step is skipped and the recovered target‒DNA complex is incubated with fresh target followed by partitioning	▪ Decrease the selection time (~1 h) ▪ No PCR step required ▪ Produce high-affinity aptamers	Developed a technique to reduce the selection time while producing aptamers of a 4-fold affinity improvement over the CE-SELEX	2006	[52]
SPR-SELEX	A method that couples the aptamer or the target to a chip then immobilizes the target or the aptamer and measures the change at the surface upon aptamer-target binding	▪ Generate aptamer molecule capable of monitoring molecular interactions between oligonucleotide sequences and its target ▪ Eliminate non-specific interaction	Provided a method to measure the aptamer’s real-time binding	2006	[53,54]
TECS-SELEX	Uses modified cells expressing recombinant form of a surface protein that eliminates the need for the purification step	Produce aptamer against ectopic cell surface protein	Produced aptamer against TGF-β type III receptor that is ectopically expressed on CHO cells	2006	[55]
▪ Genomic SELEX ▪ Transcriptomic SELEX	▪ *Genomic SELEX:* uses nucleotide library, target proteins, and metabolites from the own organism’s genome ▪ *Transcriptomic SELEX:* uses cDNA as a library	Provide a method to study in vivo oligonucleotide‒protein binding	▪ *Genomic SELEX:* identified aptamer that binds and interacts with the global regulator protein Hfq ▪ *Transcriptomic SELEX:* identified aptamer that binds to HEXIM1 protein	2006 2012	[56,57]
DeSELEX and Convergent selection	Complex SELEX methods where many proteins targets are incubated with the library and then deselecting the dominant-protein bound aptamer and redirecting the selection toward the less abundant protein/s	Shift the selection process toward a specific protein (even a less abundant one) in mixture of different protein complexes	▪ Selected aptamer against rare proteins (e.g., procoagulant proteins Factor IX and Factor VII) present in a protein mixture ▪ Demonstrated the ability to design aptamers against any protein in the proteome	2007	[58]
MonoLEX	▪ One-step selection method ▪ Separation of aptamer-target bound complex using affinity chromatography	▪ Minimize SELEX time ▪ Produce high-affinity aptamer	▪ Improved aptamer selection through skipping the amplification step ▪ Produced aptamer against the Vaccinia virus, which can also be recognized by other orthopox viruses	2007	[59]
NanoSelection^®^ (nM-AFM SELEX)	▪ Combines AFM and fluorescence microscopy ▪ Uses a binary library of a previously isolated aptamer against the target and nonsense oligonucleotide	▪ Minimize the selection cycles into one ▪ Minimize the selection time ▪ Small oligonucleotide library can be used	Developed a method to avoid multiple selection cycles and also select aptamers from small library	2007	[60]
ASExp	Uses quenching and dequenching of aptamers	Rapid selection method	Provided a method to rapidly select aptamer for different types of targets	2008	[61]
FACS-SELEX	Combines in vitro selection with FACS to separate specific cell population	▪ Target specific subpopulation of the cells ▪ Target suspended cells or primary cells	Produced aptamer bound to vital Burkitt lymphoma cells	2008	[62,63]
Single microbead SELEX	Reduces the amount of the target molecules and expose them to a single microbead	Produce high affinity aptamer using a single target-conjugated microbead	▪ Produced high affinity aptamer toward botulinum neurotoxin ▪ Feasibility of monitoring the dissociation constants of each enrichment cycle ▪ Reduced selection rounds	2008	[64]
CLADE	In silico aptamer selection method	Rapid aptamer designing method	Identified aptamers against allophycocyanin in silico	2008	[65]
In silico selection	In silico secondary structure-based selection followed by a 3D structure prediction and HTP screening of the selected aptamers	▪ Select sequence of high potential binding ▪ Reduce the library size by four to five orders of magnitude ▪ Minimize SELEX time ▪ Select high-affinity aptamers	Provided a computational approach to select aptamers of high-affinity	2009	[66]
Tissue slide-based SELEX	In situ SELEX method against paraffin tissue sections	Target clinical tumour markers and provide a way to differentiate between the clinical specimens	▪ Produced specific aptamers tightly bind to ductal carcinomas ▪ Produced specific aptamers for cancer diagnosis	2009	[67]
Sol–gel SELEX	▪ SELEX-on-a-chip selection of aptamer against multiple targets through immobilizing the protein in sol‒gel arrays ▪ Selective elution triggered by micro-heating	Produce faster, HTP, efficient, and cheap aptamer	▪ Produced high-affinity aptamer against immobilized protein (yeast transcription factor IIB protein and recombinant yeast TATA binding protein) ▪ Reduced the selection rounds ▪ HTP generation of multiple aptamers in single cycle	2009	[68]
▪ Bind-n-Seq ▪ Massively parallel SELEX ▪ Multiplexed massively parallel SELEX ▪ SELEX-seq	▪ Uses next-generation sequencing and extracts motifs from the sequence ▪ *SELEX-seq* combines SELEX with massively parallel sequencing	▪ HTP identification of multiple transcription factors (studying protein‒DNA interaction) ▪ *SELEX-seq:* determine the relative DNA binding affinity to any transcription factors	▪ Identified DNA-binding domains of two zinc-finger proteins: Zif268 and Aart ▪ Provided in vitro method to analyse protein‒DNA interaction ▪ *SELEX-seq:* identified the recognition properties of the DNA upon binding with Extradenticle–Homothorax	2009 2010 2011	[69,70,71]
M-SELEX	Target‒DNA sample separation is obtained through applying a high voltage	▪ Produce high-affinity and high-specificity aptamers ▪ Generate aptamer with low *K_d_* ▪ Select highest-affinity aptamers on chip ▪ Rapid selection	Provided a fast tool for screening aptamers against any targets	2009	[72,73,74,75]
QSAS	Combines M-SELEX with HTS for efficient and rapid aptamer production	Generate aptamer with high efficiency, high affinity, and high specificity	Introduced an integrated method for rapid, high-affinity, and high-specificity generation of aptamer	2010	[76]
Cellular-uptake in vivo-variant	Intravenous injection of modified random library into cancerous animal model	Select aptamer for internal target inside the organism depending on cellular uptake	Provided a unique approach to generate aptamer that can specifically localize to tumour cells in vivo	2010	[77]
SOMAmers	▪ Increases the physicochemical diversity of the library through incorporating chemically modified nucleotides ▪ Transforms the protein concentration signature into aptamer concentration signature that can be quantified using DNA microarray	▪ Select high-affinity aptamer of very slow off-rate ▪ Detect hundreds of proteins (large-scale proteome analysis) in small sample volumes	▪ Provided a new tool for screening biomarkers ▪ Identified 813 proteins with high sensitivity (low detection limit of ~1 pM) ▪ Identified two biomarkers for chronic kidney disease	2010	[78]
ISM	In silico post-SELEX genetic algorithm performed to identify aptamers of high-binding affinity	Improve aptamers binding-affinity and specificity	Selected aptamers of high binding affinity and specificity to prostate specific antigen, VEGF, and Proteus mirabilis	2010 2012 2013 2014	[79,80,81,82]
μFFE	Applies electric field separation to overcome the limitations of CE-SELEX to separate bound from unbound sequences	▪ Increase separation efficacy ▪ Generate higher yield from small oligonucleotide library ▪ Target immobilization is not required ▪ No need for negative selection ▪ Decrease the selection time	Improved the library size by a 300-fold over what CE-SELEX has achieved	2011	[83]
HTS-SELEX	One round of positive selection followed by HTS and informatic analysis	▪ Produce high-affinity aptamer in one positive selection round ▪ Does not require tedious work	Identified aptamers that can bind to thrombin in nM range	2011	[84]
One-step selection Method	One-step selection of target-immobilized coverslip followed by library binding, extensive washing, and amplification step	▪ One-step rapid selection method ▪ Has low binding affinity	Provided a rapid one-step generation of aptamer against α-bungarotoxin	2012	[85]
Cell-internalization SELEX	Combines cell-SELEX with HTS and bioinformatics	Produce aptamers of high intracellular internalization	Provided a new approach to design aptamers that capable of internalizations into the cytoplasm of vascular smooth muscle cells	2012	[86]
Capture-SELEX (FluMag-based)	Immobilization of specific aptamers library on magnetic beads using a docking sequence linked to the library	▪ Select aptamer against small organic molecules ▪ Select aptamers for solute targets	Provided a method to select aptamer against small molecule (e.g., kanamycin A)	2012	[87]
Domain targeted SELEX	Uses recombinant protein containing chemokine domain (to extend protein accessibility) immobilized onto magnetic agarose beads	Select aptamer against specific protein’s unfolded domain	Developed aptamer to Fractalkine protein	2012	[88]
▪ GO-SELEX ▪ Immobilization free SELEX	Uses π-π stacking and adsorption of ssDNA on 2D GO sheets to separate unbound DNA	Generation of high-affinity, cost-effective, and target-immobilization-free SELEX	Produced aptamer to Nampt protein without target immobilization	2012	[89]
MAI-SELEX	A selection method to recognize two separate sites on the target using 2’-fluoro-modified library	Target distinct sites/subunits of the protein	Produced two aptamers that recognize the αV or β3 subunits of integrin αVβ3	2012	[90]
RAPID-SELEX	▪ Skips PCR amplification step ▪ Uses affinity microcolumns	Reduce selection time	Identified aptamer to CHK2 and UBLCP1 in one-third of the time required for the conventional selection	2013	[91]
Expanded genetic alphabet (Ds-base)	Uses a library containing natural nucleotides and other three unnatural nucleotides with a hydrophobic base	Increase sequence diversity and so expand SELEX selection power	Generated aptamers that bind to VEGF165 and interferon-γ with more than 100-fold improved affinity	2013	[92]
AEGIS-SELEX	Uses unnatural forms of nucleotides, nonstandard P and Z, beside the normal four bases-based library (GACTZP DNA library)	▪ Increase sequence diversity and so expand the selection power ▪ Generate high-affinity aptamer capable of binding to hydrophobic cavities of the protein ▪ Generate aptamers of small *K_d_*, higher sequence variation, and multiple folds	Produced ZAP-2012 aptamer that can bind to MDA-MB-231 with 10-fold higher affinity than achieved using the conventional aptamers	2014	[93]
ES-SELEX	▪ Directly selects aptamer for the target protein subunit in its native structure ▪ Uses specific target competitors to elute the aptamer already bound to the target	Generate epitope-specific aptamer of a native protein structure	Produced anti-sialic acid receptor aptamers that inhibit hemagglutination at pM range	2014	[94]
MARAS	Selects aptamers with different affinities to the target molecule using magnetic beads and external rotating magnetic field	Select aptamers based on their different binding affinities	Produced aptamer with high affinity to C-reactive protein	2014	[95]
Particle Display	Measures the affinity of each aptamer sequence in the library and sorting them using HTS. Then isolating the highest-affinities candidates using FACS	Generate high-affinity, simple, rapid, and cost-effective aptamers	Measured the affinity of 100 million aptamers and obtained high-affinity aptamers for thrombin, ApoE, PAI-1, and 4-1BB proteins	2014	[96]
MSD-SELEX	Uses a library of monoclonal DNA-displaying beads	Generate high-affinity and rapid aptamers	Obtained high-affinity aptamers against EpCAM and aflatoxin B1	2014	[97]
Yeast surface display-SELEX	Target protein is loaded on the surface of yeast	Quick and unexpansive HTS identification of DNA-binding sites of the proteins without prior knowledge of the target site	Provided a way to determine aptamer‒protein binding specificity	2014	[98]
Hi-Fi SELEX	Introduces fixed-region blocking elements to enhance the functional diversity of the library	▪ Provide a functional diverse library ▪ Eliminate non-specific sequences remaining through the selection process ▪ Decrease the selection rounds	Identified aptamers of *K_d_* in nM range to α-thrombin	2015	[99]
Click-SELEX	Uses copper(I)-catalysed alkyne–azide cycloaddition modified nucleic acid libraries	Generate modified nucleobases that increase binding capabilities	▪ Provided a new method to produce novel aptamers called “clickmers” that can specifically recognize C3-GFP ▪ Increased the recognition properties	2015	[100,101]
Icell SELEX	▪ Produces aptamers to cell surface proteins using isogenic cell lines by manipulating them for positive-selection and counter-selection ▪ Controls isogenic cell protein expression by silencing (during counter-selection) and overexpression (during positive-selection)	Target various membrane proteins through successful manipulation of the endogenous expression of the target proteins	Produced aptamer to integrin alpha V	2016	[102]
LIGS	Uses antibody bound to antigen for the partitioning step to compete with aptamers from partially enriched SELEX	Identify highly-specific aptamer sequences that outcompeting with specific antibody for selecting highly specific aptamers	Identified three aptamers that outcompete with mIgM antibody	2016	[103]
Colorimetric dye-displacement SELEX	Uses target-induced displacement of a small-molecule dye to isolate signal-producing aptamers triggered by binding to the target	Develop an efficient and rapid way to isolate small molecules through colorimetric dye-displacement	Provided a new way of sensitive target isolation based on Cy7-displacement colorimetric assay	2018	[104]
Open qPCR SELEX	Uses open qPCR to quantify target-aptamer binding	▪ Simple, efficient, and low-cost selection method ▪ Avoid under- or over-amplification steps	Produced aptamers against whole Drosophila C virus particles	2018	[105]

Abbreviations: AEGIS-SELEX, artificially expanded genetic information systems SELEX; AFM, atomic force microscopy; ASExp, aptamer selection express; CE-SELEX, capillary electrophoresis SELEX; CLADE, closed loop aptameric directed evolution; ECEEM, equilibrium capillary electrophoresis of equilibrium mixtures; EMSA-SELEX, electrophoretic mobility shift assay SELEX; ES-SELEX, epitope-specific SELEX; FACS-SELEX, fluorescence-activated cell sorting SELEX; GO-SELEX, graphene oxide SELEX; Hi-Fi SELEX, high-fidelity SELEX; HTS, high-throughput sequencing; HTP, high-throughput; Icell SELEX, isogenic cell SELEX; ISM, in silico maturation; LIGS, ligand-guided selection; MAI-SELEX, multivalent aptamer isolation; MARAS, magnetic-assisted rapid aptamer selection; μFFE, micro free-flow electrophoresis; MP, minimal primer selection; MSD-SELEX, monoclonal surface display SELEX; M-SELEX, microfluidics SELEX; NECEEM, non-equilibrium capillary electrophoresis of equilibrium mixtures; PF, primer-free selection; QSAS, quantitative selection of aptamers through sequencing; RAPID-SELEX, RNA aptamer isolation via dual-cycles SELEX; SELEX, systematic evolution of ligands by exponential enrichment; SOMAmers, slow off-rate modified aptamer; SPR, surface plasmon resonance; SweepCE, sweeping capillary electrophoresis; TECS-SELEX, target expressed on cell surface SELEX.

**Table 2:** Comparison between aptamers and antibodies.

	Aptamer	Antibodies
Molecular weight	Small (~12–30 kDa)	Relatively big (~150–180 kDa)
Secondary structures	Various structures: hairpin, loop, G-quadruplex, etc	β-sheets
Generation time	Few hours to months	Several months (~six months)
Batches variations	Low	High
Immunogenicity	Low	High
Minimal target size	Targets small sizes ~60 Da	~600 Da
Targets	Wide range of targets	Immunogenic molecules
Shelf life	Long	Short
Allowed chemical modifications	Various modifications	Limited modifications
Nuclease degradation	Sensitive	Resistant
In vivo half-life	Short (~20 min)	Long (~one month)
Stability	Very stable	Sensitive to temperature and pH changes
Cost	Lower	Higher

## Abbreviations

NAs	nucleic acids
SELEX	systematic evolution of ligands by exponential enrichment
STED	stimulated emission depletion microscopy
SMLM	single-molecule localization techniques
PET	positron-emission tomography
Cryo-EM	cryo-electron microscopy
STORM	stochastic optical reconstruction microscopy
